# Socioeconomic differences in the health behaviour of children and adolescents in Germany. Results of the cross-sectional KiGGS Wave 2 study

**DOI:** 10.17886/RKI-GBE-2018-072

**Published:** 2018-06-27

**Authors:** Benjamin Kuntz, Julia Waldhauer, Johannes Zeiher, Jonas D. Finger, Thomas Lampert

**Affiliations:** Robert Koch Institute, Department of Epidemiology and Health Monitoring

**Keywords:** SOCIOECONOMIC STATUS, PHYSICAL ACTIVITY, DIET, HEALTH MONITORING, KIGGS

## Abstract

Childhood and adolescence are key determining stages for health behaviour in the life course. Frequently, health-related attitudes and patterns of behaviour that develop at young age are also maintained at adult age. As studies show, already during childhood and adolescence, patterns of health risk behaviour are more common in certain population groups. KiGGS Wave 2 results confirm that 3- to 17-year-old children and adolescents from families with low socioeconomic status (SES) eat a less healthy diet, do fewer sports and are more often overweight or obese than their peers from more affluent backgrounds. Whereas socioeconomic differences appear to have little effect on levels of alcohol consumption among 11- to 17 year-olds, girls and boys with low SES smoke more frequently than their peers with high SES. Prevention and health promotion encourage children and adolescents to adopt healthy lifestyles, and aim to drive structural changes to stimulate behaviour which promotes good health. Combining measures that target individual behaviour and a settings-based approach appears to be the most promising preventative approach to reduce health inequalities among young people. Due to the clear impacts of socioeconomic differences on health behaviour already at young age measures for disadvantaged children and adolescents and their living conditions should be given an even stronger focus in the future.

## 1. Introduction

For public health measures of prevention and health promotion, childhood and adolescence are particularly appropriate life stages [1, 2]. Health-related attitudes and patterns of behaviour that develop at young age are often maintained at adulthood (‘early determination’) [3, 4]. Correspondingly, childhood and adolescence have great significance for the promotion of healthy lifestyles. This fact also reflects in the national health targets ‘Grow up healthy: life competence, physical activity, nutrition ’ [5], ‘Reduce tobacco consumption’ [6] and ‘Reduce alcohol consumption’ [7] and in their particular focus on the young generation. Furthermore, the health-related targets of Germany’s sustainability strategy aim to stop the spread of tobacco consumption and obesity among children and adolescents [8].

**KiGGS Wave 2**

Second follow-up to the German Health Interview and Examination Survey for Children and Adolescents

**Data owner:** Robert Koch Institute

**Aim:** Providing reliable information on health status, health-related behaviour, living conditions, protective and risk factors, and health care among children, adolescents and young adults living in Germany, with the possibility of trend and longitudinal analyses

**Study design:** Combined cross-sectional and cohort study

**Cross-sectional study in KiGGS Wave 2**

**Age range:** 0-17 years

**Population:** Children and adolescents with permanent residence in Germany

**Sampling:** Samples from official residency registries - randomly selected children and adolescents from the 167 cities and municipalities covered by the KiGGS baseline study

**Sample size:** 15,023 participants

**KiGGS cohort study in KiGGS Wave 2 Age range:** 10-31 years

**Sampling:** Re-invitation of everyone who took part in the KiGGS baseline study and who was willing to participate in a follow-up

**Sample size:** 10,853 participants

**KiGGS survey waves**

► KiGGS baseline study (2003-2006), examination and interview survey
► KiGGS Wave 1 (2009-2012), interview survey
► KiGGS Wave 2 (2014-2017), examination and interview survey

More information is available at

www.kiggs-studie.de/english

Patterns of behaviour relevant to health develop through individual life experiences, knowledge and beliefs. They are also, however, related to material, structural and cultural factors, as well as historic contexts and traditions. Initially, family background and the social environment a child grows up in, influence health behaviour. As role models, parents play a particularly important role in the health behaviour of their children, in particular during their early years [9]. Parent food purchasing and consumption patterns, for example, define the family’s eating habits. Parents also provide feedback to a child’s natural desire for physical activity, either by encouraging or blocking it. Their health attitudes and preferences, as well as consumption patterns are thereby often, at least in part, adopted by their children.

As they get older, children and adolescents become more detached from their parents and begin to take independent health-related decisions, which can also be influenced by their peers [2, 9]. This applies, for example, to the use of psychoactive substances that many adolescents try and then either give up or maintain [10]. Besides the family, further environments and places with social interaction such as day care centres, schools, clubs and associations, as well as friends can influence the health behaviour of children and adolescents [11]. Yet for tobacco consumption, for example, family background does appear to weigh heavy. Studies reveal that adolescents whose parents and/or siblings smoke, smoke cigarettes and consume other tobacco products far more often themselves [12-14].

Socio-epidemiological studies indicate that child and adolescent health behaviour is affected by age and gender, and that social background also has an impact [15-17]. Consequently, socioeconomically disadvantaged children and adolescents are more likely to have an unhealthy diet [18], do less sport and a greater number of them will be either overweight or obese [19, 20] than girls and boys of the same age from more affluent backgrounds. Tobacco consumption too shows a social gradient: adolescents with low socioeconomic status (SES) smoke more frequently than those with high SES [21]. As child and adolescent health behaviour patterns are conceivably maintained at adult age and, in the long-term, play a role in the development of socioeconomic differences in morbidity and mortality [22, 23], reducing them makes a significant contribution to reducing unequally distributed health opportunities.

Developing and evaluating measures to close the social gradient in the health-relevant behaviour of children and adolescents requires regular, reliable and robust data. Based on the cross-sectional data from the German Health Interview and Examination Survey for Children and Adolescents (KiGGS Wave 2, 2014-2017), this article provides an overview of the current extent of socioeconomic differences in the health behaviour of children and adolescents.

## 2. Methodology

### 2.1 Study design and sample

KiGGS is part of the health monitoring system at the Robert Koch Institute (RKI) and includes repeated representative cross-sectional surveys for Germany of children and adolescents aged 0 to 17. Whereas the KiGGS baseline study (2003-2006) was designed as an examination and interview survey, the first follow-up survey (KiGGS Wave 1, 2009-2012) was conducted as an interview-based survey by telephone. KiGGS Wave 2 (2014-2017) again collected examination and interview data, whereas, unlike the KiGGS baseline study, many participants were only interviewed and not examined. The concept and design of KiGGS have already been described [24-27]. A total of 15,023 respondents (7,538 girls and 7,485 boys) took part in KiGGS Wave 2 (response rate 40.1%). 3,567 children and adolescents were examined (1,801 girls and 1,766 boys) (response rate 41.5%).

### 2.2 Indicators

This article analyses four areas of health-relevant behaviour in childhood and adolescence: diet, physical activity, body mass index and substance use. For each of these four areas two exemplary indicators were analysed, the majority of which were included as Fact sheets in issue 1/2018 of the Journal of Health Monitoring. Family socio-economic status (SES) serves as an independent variable; its operationalisation has also already been described in detail in issue 1/2018 of the Journal of Health Monitoring [28].

#### Diet

In KiGGS Wave 2 – like in the KiGGS baseline study – the consumption of selected food items was assessed with a food frequency questionnaire [29, 30]. Amongst others, the questionnaire collected data on fresh fruit intake and consumption of sugary soft drinks (Cola, lemonade, ice tea, malt beer and energy drinks) ‘during the past four weeks’. There was a total of eleven answer categories, stretching from ‘never’ to ‘more than five times per day’. Parents (or guardians) answered the questions for the group of 3- to 10-year-olds, children and adolescents aged 11 to 17 answered themselves [30]. This article presents the proportion of children and adolescents who ate fresh fruit or consumed sugary soft drinks daily during the last four weeks.

#### Physical activity

Data on physical activity (including sports) was collected in KiGGS Wave 2 by self-reporting (11- to 17-year-olds) or based on the answers of guardians (3- to 10-year-olds) in a written questionnaire [31]. Levels of physical activity were defined based on the following question: ‘On how many days of a normal week are you/is your child physically active for at least 60 minutes on a single day?’ The eight answer categories spanned from ‘On no day’’ to ‘on seven days’. The present analyses are based on the recommendations of the World Health Organization (WHO), which recommends at least 60 minutes of moderate- to vigorous-intensity physical activity daily [32]. The question: ‘Do you/does your child do sports?’ measured sport activity. A comment was included stating that: ‘This covers all kinds of sport, in or outside of a club, except for sports at school and/or sport activities in kindergarten’. The present analysis shows the proportion of children and adolescents who do sports during leisure time.

#### Body mass index

In KiGGS Wave 2 body height and weight of respondents aged 3 to 17 were measured by applying a standardised procedure in line with the baseline study [33]. A person’s body mass index (BMI) was calculated from the ratio between body weight and height (kg/m^2^). Since the relationship between body height and weight changes during childhood and adolescence due to growth, there is no uniform cutpoint for all age groups from which a child or adolescent is classified as overweight or obese. For this reason, up to the age of 18 year, BMI percentile curves are applied which reflect BMI distribution with regard to a reference population and take age and gender into account. In Germany, overweight and obesity are usually defined based on the recommendations of the Arbeitsgemeinschaft Adipositas im Kindes- und Jugendalter (AGA), and by applying national reference percentiles according to Kromeyer-Hauschild et al. [34, 35]. Children with a BMI above the 90th percentile are considered overweight and obesity is defined as a BMI above the 97th percentile.

#### Substance use

In KiGGS Wave 2 substance use was only measured in the 11- to 17-age group. Participants responded in writing to questions about smoking behaviour and alcohol consumption [11]. Respondents answered the question, ‘Do you currently smoke?’ by choosing between one of the following answers: ‘No’, ‘Daily’, ‘Several times per week’, ‘Once per week’ and ‘Less than once per week’. All respondents who stated that they smoke tobacco – including only occasionally – are grouped as current smokers [36]. The question ‘Have you ever drunk alcohol?’ (answer categories ‘Yes’ and ‘No’) measured lifetime prevalence of alcohol consumption.

#### Socioeconomic status

In KiGGS Wave 2 the socioeconomic status (SES) was measured through an index based on the information parents provided on educational background, occupational status and income situation (equivalised disposable income) [28]. The operationalisation applied corresponds to the KiGGS Wave 1 approach [37]. For the purpose of analysis, the three groups of low, medium and high status were established, with the low and high status group each comprising of around 20% and the medium status group of around 60% of the study population [28].

### 2.3 Statistical analysis

In the fields of diet and physical activity, the analyses are based on the data of 13,568 respondents (6,810 girls, 6,758 boys) aged 3 to 17, for substance use on the data of 6,599 respondents (3,423 girls, 3,176 boys) aged 11 to 17. For certain indicators, a varying number of respondents were excluded from the analyses because they did not provide all the necessary answers. The analysis of BMI values is based on the data of 3,561 adolescents (1,799 girls, 1,762 boys) aged 3 to 17 with valid answers on body height and weight. The results are stratified by gender and socioeconomic status (SES) based on prevalence with a 95% confidence interval (CI 95%). Moreover, adjusted odds ratios (aOR) with 95% confidence intervals are provided that indicate the factor by which the statistical probability is increased for a certain be haviour to be present in the low or medium status groups compared to the high status group defined as reference category. The underlying logistic regression analysis statistically controls structural differences in the composition of status groups regarding age, gender and family migration background [38].

To achieve representative data, the calculations were carried out using a weighting factor that corrected for deviations within the sample from the population structure with regard to age in years, gender, federal state, German citizenship (as of December 31 2014) and the parents level of education based on the Comparative Analysis of Social Mobility in Industrial Nations (CASMIN) [39] (Microcensus 2013 [40]). A specific weighting factor, i.e. one which is related to the examination participants, was applied to measurement results for overweight and obesity.

All analyses applied Stata 14.2 to the KiGGS Wave 2 data set (Version 5) (Stata Corp., College Station, TX, USA, 2015). To adequately account for the clustering of participants at sample points and weighting in the calculation of confidence intervals and p-values, Stata survey commands were used [41]. A statistically significant difference between groups is assumed to have been demonstrated among groups with p-values of less than 0.05.

## 3. Results

### 3.1 Diet

**Figure 1:**
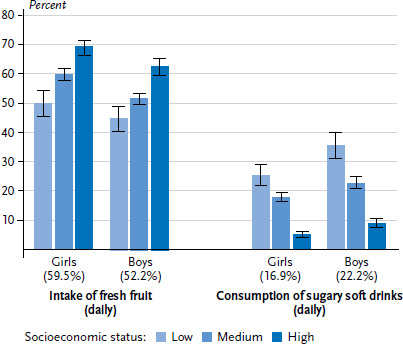
Dietary habits of 3- to 17-year-olds according to gender and socioeconomic status (Fruit n=6,473 girls, n=6,375 boys; Sugary soft drinks n=6,467 girls, n=6,372 boys) Source: KiGGS Wave 2 (2014-2017)

According to the results of KiGGS Wave 2, more than half (55.8%) of all children and adolescents aged 3 to 17 in Germany eat fresh fruit daily. Girls eat fresh fruit daily more often than boys (59.5% vs. 52.2%). With increasing age the proportion of girls and boys eating fresh fruit every day decreases. Regardless of gender, a higher SES translates into a greater proportion of children and adolescents who eat fresh fruit daily (Figure 1). Whereas only 47.2% of children and adolescents with low SES eat fresh fruit daily, the rate for children and adolescents with medium SES is 55.7% and, at 65.4%, significantly higher in particular for those with high SES.

Around one fifth (19.6%) of 3- to 17-year-old children and adolescents in Germany drinks sugary soft drinks daily – boys (22.2%) significantly more often than girls (16.9%) [30]. With age, the proportion of girls and boys who drink sugary soft drinks daily increases. Moreover, the results confirm a pronounced social gradient: the proportion of children and adolescents, who drink sugary soft drinks daily is higher the lower their SES [30]. Whereas nearly one third (30.5%) of children and adolescents with low SES drinks sugary soft drinks daily, it is around one fifth (20.2%) in the medium SES group and a mere 7.1% of children and adolescents from the high SES group. These pronounced differences are evident in both genders (Figure 1).

### 3.2 Physical activity

**Figure 2:**
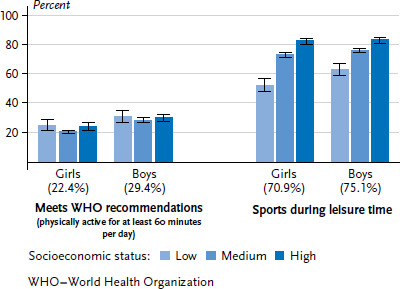
Physical activity (including sports) among 3- to 17-year-olds according to gender and socioeconomic status (Physical activity n=6,469 girls, n=6,394 boys; Sports n=6,504 girls, n=6,413 boys) Source: KiGGS Wave 2 (2014-2017)

Around one quarter (26.0%) of 3- to 17-year-old children and adolescents in Germany is physically active for at least 60 minutes on every day of a normal week and thus fulfils the WHO recommendations for physical activity [31]. The proportion for boys (29.4%) is higher than for girls (22.4%). With increasing age, the share of girls and boys who meet the WHO recommendations for physical activity gradually decreases. For the physically active, family SES appears not to make any significant difference to girls or boys (Figure 2). For the physically inactive (defined as physically active for at least 60 minutes on fewer than two days per week), however, pronounced socioeconomic differences are apparent, with a greater proportion of girls and boys with low SES in this group than of girls and boys with medium and high SES (data not shown, see [31]).

Nearly three quarters (73.0%) of 3- to 17-year-old children and adolescents in Germany do sports during their leisure time – boys (75.1%) slightly more than girls (70.9%). The highest levels of sports activities are registered in the 7- to 13-age-group. The higher the SES, the higher the proportion of children and adolescents who do sports during leisure time. 58.0% of children and adolescents with low SES do sports, around three quarters (74.6%) of those with medium SES and 83.1% of those with high SES. Such a pronounced social gradient is apparent for both girls and boys (Figure 2).

### 3.3 Body mass index

**Figure 3:**
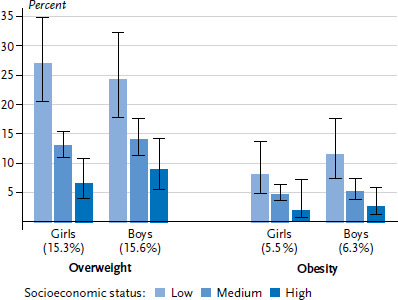
Overweight and obesity among 3- to 17-year-olds according to gender and socioeconomic status (n=1,733 girls, n=1,704 boys) Source: KiGGS Wave 2 (2014-2017)

The values measured for body height and weight in KiGGS Wave 2, indicate that, based on the reference values published by Kromeyer-Hauschild et al. 2015 [34], 15.4% of 3- to 17-year-old children and adolescents in Germany are overweight [33]. Obesity prevalence is at 5.9%. No significant gender differences are apparent in overweight and obesity prevalence figures. For both genders, however, the proportion of overweight and obese children and adolescents rises with age. Figures for overweight reveal a social gradient, with a lower SES correlating to a higher proportion of overweight children and adolescents (Figure 3). Whereas a total of around one quarter (25.5%) of 3 to 17-year-olds in the low status group is overweight, the same applies for only one in around seven children (13.5%) in the medium status group and one in thirteen (7.7%) in the high status group. The proportion of obese children, too, is also significantly higher in socioeconomically disadvantaged families than in more affluent families (low SES 9.9%, medium SES 5.0%, high SES 2.3%) (Figure 3).

### 3.4 Substance use

**Figure 4:**
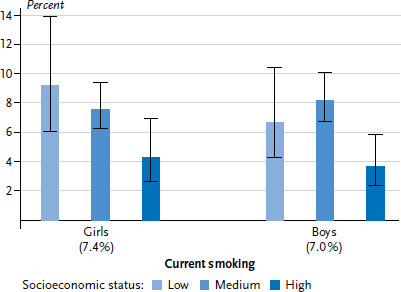
Current smoking among 11- to 17-year-olds according to gender and socioeconomic status (n=2,949 girls, n=2,702 boys) Source: KiGGS Wave 2 (2014-2017)

**Figure 5:**
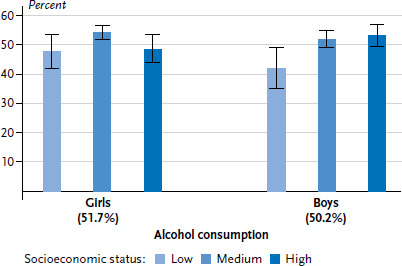
Alcohol consumption (lifetime prevalence) for 11- to 17-year-olds according to gender and socioeconomic status (n=3,165 girls, n=2,876 boys) Source: KiGGS Wave 2 (2014-2017)

Based on KiGGS Wave 2 data, 7.2% of 11- to 17-year-olds smoke at least occasionally – with in total only small differences between girls and boys [11, 36]. For both genders, smoking prevalence increases with age. Overall, smoking rates for adolescents from low (8.0%) and medium (7.9%) SES backgrounds is around twice as high, compared to those of high SES background (4.0%). For girls, the most pronounced difference was registered between the low and high status groups, for boys between the medium and high status groups (Figure 4). In KiGGS Wave 2, around half (51.0%) of 11- to 17-year-old adolescents stated that they had drunk alcohol at least once. Whereas the proportion for girls (51.7%) and boys (50.2%) is nearly equal, the lifetime prevalence of alcohol consumption increases, as can be expected, with age [11]. Overall, the proportion of 11- to 17-year-olds who have drunk alcohol at least once is lower for those with low SES (44.9%) than for those with medium (53.2%) or high SES (51.1%). Differentiated by gender, a lower lifetime prevalence of alcohol consumption is only evident for boys with low SES, whereas no such significant difference between status groups is found for girls (Figure 5).

### 3.5 Multivariate results

Multivariate analyses indicate that even when statistically controlling for the differences in status group composition regarding age, gender and family migration background, children and adolescents with low SES generally show higher levels of risky health behaviour than their more affluent peers (Table 1).

Regarding diet, for example, compared to the high SES reference group, the odds of eating fresh fruit daily, is only half as high for those with low SES (aOR 0.48 (0.41-0.56)), whereas the probability of consuming sugary soft drinks daily is increased by a factor of about 6 (aOR 5.91 (4.87-7.19)). As regards physical activity, the findings are less clear. No significant differences in levels of physical activity can be found between status groups (based on the WHO recommendations: at least 60 minutes of physical activity daily) (aOR 1.12 (0.92-1.35)). However, the odds of doing sports during leisure time and outside of kindergarten and school is significantly lower for children and adolescents with low SES compared to those with high SES (aOR 0.29 (0.24-0.34)). Data on body height and weight and the corresponding BMI values evidence that the risk of being overweight (aOR 3.44 (2.13-5.55)) or obese (aOR 4.26 (1.76-10.31)) is around three to four times as high for children and adolescents with low SES compared to those with high SES. Regarding substance use, the results for the relation between tobacco and alcohol consumption and SES differ. While the results on lifetime prevalence of alcohol consumption in 11- to 17-year-olds show a lower risk for children and adolescents with low SES (aOR 0.65 (0.47-0.89)), results on tobacco consumption show that children and adolescents with low SES smoke around twice as often as those with high SES (aOR 2.06 (1.20-3.51)).

For the majority of indicators considered, both children and adolescents with low SES, and also those with medium SES, far more frequently show risky health behaviour compared to their peers with high SES (Table 1). For some indicators, such as sugary soft drink consumption or leisure time sports activities, multivariate results moreover indicate a marked social gradient, with a higher SES being associated with a lower risk for risky health behaviour and/or a higher likelihood of behaviour which promotes good health. With a few notable exceptions, socioeconomic differences impact on the health behaviour of girls and boys in a very similar way. One such exception is the lifetime prevalence of alcohol consumption: whereas for girls the differences between status groups are not significant (aOR 0.83 (0.51-1.36)), among boys, those with low SES are less likely to have drunk alcohol at least once, compared to their peers with high SES (aOR 0.52 (0.34-0.81)).

## 4. Discussion

Health-relevant behaviour plays a fundamental role in the development and course of chronic diseases. KiGGS Wave 2 results indicate that socioeconomic differences already become apparent in health behaviour during childhood and adolescence. Socioeconomically disadvantaged children and adolescents eat less healthy food, do less leisure time sports activities and are more prone to being overweight or obese; and they smoke more often compared to their more affluent peers. The only areas, where no such differences to the detriment of disadvantaged children and adolescents were found, are physical activity according to the WHO recommendations, and the lifetime prevalence of alcohol consumption. The two previous KiGGS Waves – the KiGGS baseline study (2003-2006) and KiGGS Wave 1 (2009-2012) – reported similar results [42, 43]. The KiGGS results are thereby highly compatible with national and international research [15, 17]. For example, the German school entry health examinations indicate that socioeconomically disadvantaged children are signficantly more prone to being overweight or obese compared to those from more affluent families [44-46]. International comparative studies, such as the WHO-funded Health Behaviour in School-aged Children study (HBSC) [47], indicate, that in western industrialised nations, there are manifest social differences in the health behaviour of growing generations – usually to the detriment of children and adolescents from socioeconomically disadvantaged families [18, 48, 49].

When interpreting these findings, it is important to bear in mind that health-relevant behaviour should not be analysed without factoring in structural conditions and environmental determinants, which evidently influence behaviour [50, 51]. To a certain extent, such interdependencies can explain, why socioeconomically disadvantaged children and adolescents have a greater tendency towards risky health behaviour. Individual behaviour and complex behaviour patterns are only based on free choice to a limited degree. They are also always the result of a confrontation with the currently dominant living conditions [52]. For example, the probability of people being physically active in leisure time and the amount of time they spend active, also depends on their living environment (parks, playgrounds, sports offers, traffic and safety etc.). Conversely, the probability of having an unhealthy diet (in particular for people whose income is low) increases, if the offering in the neighbourhood consists mainly of fast food restaurants, in particular, when their products are cheaper than unprocessed fresh products such as fruit and vegetables. If the complex causes of health behaviour and the role played by living conditions (settings, material resources, education, environmental factors etc.) are not considered, there is a danger of one-sidedly blaming the victim i.e., that segment of the population, which is affected by the majority of health risks [53]. It will require comprehensive structural measures to improve the overall health behaviour of children and adolescents, and mitigate the role played by socioeconomic differences in the health behaviour of the growing generation. It is clear from past experiences that educational approaches and individual measures such as training sessions or courses, which merely aim to change the behaviour of individuals (prevention through lifestyle modification), have only a limited effect [54]. Moreover, there is a certain risk that socioeconomic differences in health behaviour further increase because disadvantaged population groups are not or not so easily reached by such measures (prevention dilemma) [51, 55, 56]. Demonstrably better results are achieved when behavioural prevention is supported by broader measures that target specific living conditions and/or social structures and therefore the underlying factors that influence health behaviour (settings approach). A setting approach aims to change people’s living conditions in ways that ‘make the healthier choice the easier choice’ [57]. Behavioural prevention and a setting approach are not mutually exclusive approaches, rather, they can complement each other [58]. Actually, a combination of behavioural prevention and setting approaches in the sense of a policy mix seems to be particularly promising. Several stakeholders indicate this, such as the German Alliance against Non-communicable Diseases (NCD Alliance), an association of 20 scientific medical expert panels, associations and research institutes that have been promoting sustainable and national level primary prevention in Germany since 2010 [55, 59].

After the initial results of the KiGGS baseline study became available, Germany adopted its federal government strategy to promote child health that explicitly aims to promote equal opportunities in health for children and adolescents [60]. Moreover, as part of national health targets (gesundheitsziele.de), the health and health behaviour of children and adolescents are being granted central importance. For example, the national health target ‘Grow up healthy’, created in 2003 and updated in 2010, not only promotes life competencies but also puts a focus on diet and physical activity [5]. The same applies to the national action plan IN FORM, which aims to improve dietary choices and levels of physical activity in Germany in the long-term [61]. Germany’s national recommendations for physical activity and physical activity promotion published in 2016, conclude that evidence so far is insufficient for developing recommendations to reduce the impact of socioeconomic differences on levels of physical activity among children and adolescents [54]. However, the literature indicates three types of interventions that could lead to more equal opportunities in society: 1. Interventions focusing on a settings approach, 2. Interventions targeted directly at socioeconomically disadvantaged individuals (target group orientation), 3. Interventions with the active participation of target groups in decisions regarding design and implementation (participation) [54]. Under the guidance of Germany’s Federal Centre for Health Education (BZgA), the co-operation network Equity in Health offers a comprehensive database of cases focused on promoting the health of disadvantaged children and adolescents, develops quality criteria and identifies projects of good practice to be recommended [62, 63]. In 2015, Germany adopted the Preventive Health Care Act, which provides additional resources for setting-oriented measures [64]. The Act obliges social insurance carriers, federal states and municipalities to work more closely together on matters relating to prevention and health promotion. It specifically highlights the importance of a settings-orientation to ‘determine health-relevant social systems’ (section 20 of the German social insurance code SGB, book V), which provide the framework for everyday conditions of living, learning and working. For different stages in life, the relevant settings and target groups are also different. As children and adolescents spend a great deal of time at child day care centres [65] and schools [66], these institutions are particularly appropriate settings for promoting good health. As education institutions reach children and adolescents regardless of their socioeconomic background, this also applies to the longer term goal of balancing socioeconomic differences [17].

This cross-sectional analysis’ particular strength is in sampling design, conduction and weighting which allows the results for the overall German population to be generalised. As with all surveys, a bias due to selective non-participation can however not be ruled out [25]. With the exception of values for body weight and height which are required to calculate the body mass index, the reported prevalences are based on parent- or self-reported data for the 3- to 17-year-old children and adolescents. As in other interview surveys, the degree to which socially desired responses distort the results remains unclear. The results so far cannot answer the important question as to whether the impact of socioeconomic differences on health behaviour has increased or not over the last 15 years. However, data for most of the indicators was collected similarly in the KiGGS baseline study and KiGGS Wave 1, and, corresponding trend analyses will be the next step. KiGGS cohort data, again, which includes many respondents from the KiGGS baseline study [67], provides answers on the development of socioeconomic differences in the health behaviour of children and adolescents in individual life courses. Longitudinal analyses of this cohort data can potentially help clarify how socioeconomic differences in health behaviour evolve during important life course transitions, for example from childhood to adolescence or from adolescence to emerging adulthood. Hardly any comparable studies from Germany are available so far [68].

For central fields of health behaviour (for example diet, physical exercise and substance use), an early determination of behaviour patterns can be assumed that then become relatively stable at later stages in life. From a public health point of view, this creates the challenge (and opportunity) for achieving long-term results through co-ordinated, evidence-based interventions at childhood and adolescence. Socioeconomic differences in the health behaviour of children and adolescents demand a combination of behaviour and settings-oriented prevention measures, as well as socially sensitive prevention policies [3]. Their success should always be measured in terms of the degree to which they manage to reach socioeconomically disadvantaged population groups. In addition to health policy, further policy fields should be included in line with the Health in All Policies approach in order to anchor health-related questions and the goal of health equity at all levels and spheres of politics and society [69, 70].

## Key statements

- Children and adolescents with low SES eat fresh fruit less frequently daily and consume sugary soft drinks more often daily than their peers with high SES.
- Children and adolescents with low SES meet the WHO recommendations for physical activity almost as often as their peers with high SES, but do significantly fewer sports during leisure time.
- The lower the SES of children and adolescents, the higher the prevalence of overweight and obesity.
- Whereas socioeconomic differences in alcohol consumption are less pronounced, more girls and boys with low SES smoke than their peers with high SES.
- The success of measures to promote healthy lifestyles is also reflected in whether these measures succeed in reaching socioeconomically disadvantaged children and adolescents.

## Figures and Tables

**Table 1:** Socioeconomic differences in the health behaviour of children and adolescents. Results of logistic regression controlled for age, gender and family migration background Source: KiGGS Wave 2 (2014-2017)

Indicator	Age	Girls		Boys		Total	
		SES low vs. high	SES medium vs. high	SES low vs. high	SES medium vs. high	SES low vs. high	SES medium vs. high
		aOR (95% CI)	aOR (95% CI)	aOR (95% CI)	aOR (95% CI)	aOR (95% CI)	aOR (95% CI)
**Diet**							
Daily consumption of fresh fruit during the past four weeks	3-17	**0.49**	**0.71**	**0.47**	**0.67**	**0.48**	**0.69**
		**(0.39-0.61)**	**(0.61-0.83)**	**(0.37-0.60)**	**(0.57-0.78)**	**(0.41-0.56)**	**(0.62-0.77)**
Daily consumption of sugary soft drinks during the past four weeks	3-17	**6.27**	**3.97**	**5.85**	**2.91**	**5.91**	**3.28**
		**(4.49-8.75)**	**(3.04-5.18)**	**(4.43-7.73)**	**(2.29-3.69)**	**(4.87-7.19)**	**(2.77-3.89)**
**Physical activity**							
Physical activity (physically active for at least 60 minutes per day)	3-17	1.26	0.88	1.02	0.95	1.12	0.92
		(0.96-1.66)	(0.74-1.05)	(0.78-1.32)	(0.81-1.11)	(0.92-1.35)	(0.82-1.04)
Sports during leisure time	3-17	**0.27**	**0.58**	**0.30**	**0.59**	**0.29**	**0.59**
		**(0.20-0.35)**	**(0.47-0.71)**	**(0.23-0.40)**	**(0.48-0.73)**	**(0.24-0.34)**	**(0.51-0.68)**
**Body mass index**							
Overweight (according to Kromeyer-Hauschild et al. 2015 [34])	3-17	**3.83**	**2.08**	**3.21**	1.70	**3.44**	**1.84**
		**(1.90-7.72)**	**(1.12-3.83)**	**(1.62-6.35)**	(0.91-3.18)	**(2.13-5.55)**	**(1.22-2.79)**
Obesity (according to Kromeyer-Hauschild et al. 2015 [34])	3-17	4.04	2.45	**4.40**	2.05	**4.26**	**2.23**
		(0.91-17.86)	(0.65-9.18)	**(1.50-12.91)**	(0.76-5.53)	**(1.76-10.31)**	**(1.00-4.94)**
**Substance use**							
Current smoking	11-17	**2.14**	1.71	1.97	**2.30**	**2.06**	**1.98**
		**(1.04-4.40)**	(0.98-2.98)	(0.96-4.05)	**(1.35-3.92)**	**(1.20-3.51)**	**(1.34-2.94)**
Alcohol consumption (lifetime prevalence)	11-17	0.83	1.09	**0.52**	0.79	**0.65**	0.91
		(0.51-1.36)	(0.77-1.54)	**(0.34-0.81)**	(0.60-1.03)	**(0.47-0.89)**	(0.75-1.11)

aOR=adjusted odds ratio; SES=socioeconomic status; WHO=World Health Organization; CI=confidence interval; bold=statistically significant (p<0.05)
